# An unusual presentation of appendicitis: a 23 cm long appendix in Morocco

**DOI:** 10.11604/pamj.2019.32.72.18151

**Published:** 2019-02-12

**Authors:** Hicham Laraqui, Mohamed Lamgari, Mohamed Essarghini, Aziz Zentar

**Affiliations:** 1 Department of General Surgery, Military Hospital Rabat, Faculty of Medicine, Fes, Morocco; 2 Department of General Surgery, Military Hospital, Faculty of Medicine, Mohamed V University, Rabat, Morocco

**Keywords:** Appendix, acute inflammation, long

## Abstract

The appendix is a diverticulum attached to the caecum. It can have variable lengths and locations. Acute inflammation from long appendix is a diagnostic challenge with unusual signs and symptoms. A 35 years old man admitted to the emergency department for an acute abdominal pain with low-grade fever which had been present for 03 days. The abdomen Ultrasound showed minimal effusion in the right iliac fossa. He underwent an appendicectomy which revealed a very long appendix (23 cm in length) with tip reaching the sub hepatic area. The surgeon must kip in mind all anatomical variations of the appendix for making diagnosis and decisison to operate acute appendicitis because the increasing risk of morbidity.

## Introduction

The appendix is usually 6-9 cm long but different length has been reported from 1 cm to 30 cm [1]. Acute appendicitis is the most common disease of the appendix and appendicectomy is a very frequent operation performed in emergency. Acute appendicitis from long appendix may present with unusual symptoms and atypical clinical presentation making the diagnosis very difficult.

## Patient and observation

**Figure 1:**
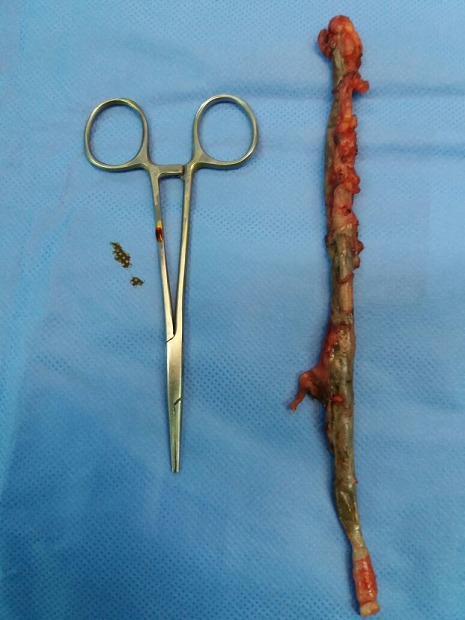
The long appendix after dissection

**Figure 2:**
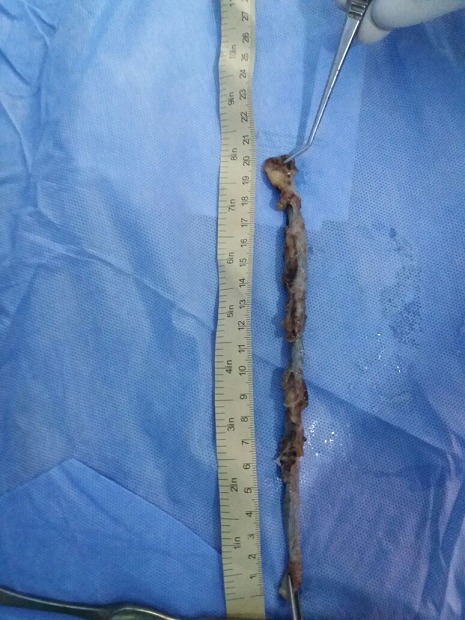
The long appendix after dissection and formalin fixation

35 years old man admitted to the emergency department for an acute abdominal pain with low-grade fever wich had been present for 03 days. The patient presented nausea, vomits and loss of appetite. Physical exam showed a deep tendress in the right iliac fossa. Blood test showed total leucocytes count of 12000/mm^3^ with neutrophil count of 68%. Others blood tests were normal. The abdomen Ultrasound showed minimal effusion in the right iliac fossa. The computed tomography (CT) scan was normal. The diagnosis of acute appendicitis was made indicated an appendicectomy. A largest Mac Burney incision was made. The appendix was inflamed with retro-ceacal position. It was very long and the tip reached the sub hepatic area. The appendix measured 23 cm in length (Figure 1). The appendicectomy was made. The pathological exam found a long appendix (20 cm after formalin fixation) (Figure 2) with ulcerative meso and lumen. The pathological report confirmed an acute appendicitis without signs of malignancy. The postoperative course was uncomplicated. The patient left hospital on post-operative day 3.

## Discussion

The appendix is a diverticulum arising from the inferior tip of the caecum; its length is 6-9 cm in adults [1]. There are a considerably variations in length and position (free tip) that can give clinical and radiological misdiagnosing. The appendix free tip may be retrocaecal, pelvic, paracolic, rarely pre, post-ilea, or sub hepatic [2, 3]. Very rarely, the appendix may occupy a left position of the abdomen in situs inversus. The length may vary between 2 cm to 30 cm [4]. The longest appendix reported in Guinness World Records measured 26 cm removed from 72 years old during an autopsy in Croatia [5]. Some author's found that the appendix length is highly correlated with body weight [6]. Our patient had an unusually long appendix of 23 cm, making it longest reported in Morocco (Base PubMed and Medline). When inflamed, longest appendices produce confusing picture making the difficulty for diagnosis. It may simulate inflammation of other structures such as enteritis, salpingitis, scrotal pain and endometriosis [7]. Sub hepatic appendix can mimic cholecystis and perforation of sub hepatic appendix can mimic liver abscess [8, 9]. These symptoms make difficulty for the diagnosis. In our case, even the appendix tip reached the sub hepatic area; the pain and tenderness were in the right iliac fossa because the inflammation was located in the appendix base. Computed tomography scan with inflammatory markers and leukocytosis can help diagnosis for acute appendicitis in atypical cases [10].

## Conclusion

Long acute appendicitis is unusual making a misdiagnosis with others inflammatory diseases. the surgeon must keep in mind all anatomical variations of the appendix for making diagnosis and the decisison to operate because the increasing risk of perforation and morbidity in negleted appendicitis. Therefore, a high index of suspicion and good clinical sense are needed to make a diagnosis of acute appendicitis in atypical cases.

## Competing interests

The authors declare no competing interests.
